# Changes in Psychopathology and Mental Health Resilience

**DOI:** 10.3389/fpsyt.2021.676492

**Published:** 2021-05-20

**Authors:** Georgi Onchev

**Affiliations:** Department of Psychiatry and Medical Psychology, Medical University Sofia, Sofia, Bulgaria

**Keywords:** psychopathology, culture, mental health, resilience, overdiagnosis

## Abstract

Accelerated culture transition and contemporary post-truth mass cognitive distortions contribute to cognitive insecurity and substitution of facts with opinions. In this paper the impact of these changes on normal psyche and on psychopathological manifestations and mental health care is described. The consequences include drop of mental resilience, pathomorphosis of some clinical pictures, blurring of diagnostic boundaries, mimicry of psychopathology, and overdiagnosis. Their repercussions on mental health care and professional integrity are discussed, and particularly the need for shift of mental health care focus from protection to resilience is disputed.

## Introduction

Clinical pictures in psychopathology change with time—and this is not unexpected. Most psychiatrists today are unfamiliar with neurolues, and most psychiatrists few generations ago were not acquainted with borderline personality disorder for instance. Current acceleration of historical times however has the potential to cause unparalleled from the past cultural and mental alterations (1), particularly in high-income countries. While one of the basic functions of culture is to serve as social DNA (2, 3) preserving basic unwritten rules and restraining abrupt changes in human communities, this acceleration may have profound effect on the way how normalcy and psychopathology are discriminated. If, simplifying, psychopathology can be viewed as deviation from a conditional norm, what is primarily affected by the change in mentality nowadays is the norm itself, i.e., the referent value against which we measure, and only secondarily—the deviation from it.

## Culture and Mentality Alterations

This change stems from contemporary cognitive distortions which are probably best characterized by inability to recognize truth, considered by the American philosopher H. Frankfurt in his influential essay *On Bullshit* (4) as a main cultural trait of modern times. He argues that resulting falsehood is more dangerous than deliberate lie because the liar recognizes (and for this reason conceals or distorts) truth, while the bullshitter substitutes it with near-truths that are more covert and misleading than frank lie. Understandable consequences include negligence to facts and to the words representing them. This depiction presents the essence of the contemporary cognitive context—substitution, e.g., of facts with opinions. Post-truth adds new shades to it with its appeal to feelings rather than to facts, and the euphemisms of political correctness create an impression for evasion of speech and for relativeness of reality testing through it. Undoubtedly, each speech is contextual, however mass disrespect to precise wording, especially in the social media, may result in equation of awareness to ignorance. Inability for orientation in news streams causes confusion, agnosticism, poor discrimination between fiction and reality (as in psychosis), and prevailing of personal views that gain own ontological status (as with magical thinking).

Late modern culture contributes to cognitive confusion with global topics like discourse, ecological concerns, fluidity of boundaries and identities, deconstruction (and reconstruction) of reality, polyphony. Related tolerance, soft skills, and virtual looseness of identities reinforce the tendency to relativisation and cognitive blurring. Parallel to the rise of mankind's affluence, values do change. Findings of the World Values Survey^1^ show that the rise of the GDP is associated with values transition from survival to self-expression, along with transition from traditional religious to the rational secular pole. And, alienation, loss of ipostas, inner emptiness, and moral over-permissiveness, are all associated with secular orientation.

Each culture nourishes own cognitive distortions. In fact, culture can be viewed as a creative distortion of reality by myths, fairytales, rites, and stories about enduring and links with the ancestors that make living more bearable (2, 5, 6). Contemporary schemas (7) however lose their adaptivity and turn to be increasingly dysfunctional as concerns the relations between the individual and the world around. The impact of these changes on normal psyche include loss of vigilance and intuitiveness, rise of consumerism and hedonism, and shared identities and even mental contents like *memes* (8). The incomparable in human history affluence is related to lack of coercion to earn bread, and even probably to a presumed from evolutionary perspective uselessness of genes that have since prehistoric times contributed to survival, and for this reason been selected (9).

## Impact on Resilience and Psychopathology

Distorted views about reality and hedonistic life styles lead to drop of mental resilience manifested in low tolerance to inconveniences and negative feelings. The mainstream view about mental health—in accord with the WHO's definition of health in general (10)—as a state of complete well-being and not merely the absence of disease or infirmity, should not be misinterpreted as exclusion of any burdens and discomfort from human experience. Such a perspective of mental health, influenced by hedonic and eudaimonic traditions championing positive emotions and excellence in functioning, as Galderisi et al. (11) determine it, risks excluding plenty of everyday human experiences. Bad feelings and healthy suffering should be included in the concept of euthymia because “life is no rose garden” (12) and negative emotions are part of everyday life (without being symptoms). Such emotions like anxiety, and probably sadness, may have even played vital role for survival in the past (9). In brief, normalcy is not perfection. And resilience (not only well-being) should be considered a vital component of it.

The current COVID-19 pandemic situation poses serious concerns about mental health and corresponding challenges in front of psychiatric services and other stakeholders to cope with isolation, stress, anxiety and grief (13). We witness however also the effect of low mental resilience in this situation: necessary limitations of personal freedoms as part of the epidemiological safety measures easily provoke anger, protest, and denial due to non-toleration of anxiety and discomfort. As a natural triage, pandemia maps and discriminates between overreactions and more mature responses, as well as between complaining and problem solving, and between strengths and deficits in health care systems or in media policies. The frequent pathologization of such overreactions as depressive and anxiety manifestations of mental health infringement encourages seeking help for minor reasons as part of the general current trend for overdiagnosis in psychiatry (14). Not to mention that escape from negative emotions (or their premature sedation) deprives the individual of the opportunity to seek meaning in them and to improve one's mental stamina.

The impact of these changes on psychopathology is many-sided (Table 1). Psychopathology is not a sum of frozen clinical pictures—and has always changed with time. Understandably, alterations in physical environment affect diseases, e.g., with drop of organic syndromes due to infectious agents (5). Likewise, alterations in cognitive landscape, cultural background, and treatments may affect delineation, presentation, and even course of the mental disorders. A resourceful study of Jablensky et al. (15) finding with the Present State Examination (PSE) and the diagnostic algorithm CATEGO good internal consistency and syndrome demarcation in 53 patients with dementia praecox and 134 with manic-depressive insanity from the 1908 archive of the Munich psychiatric clinic (at the time when Kraepelin worked there), as well as 80.2% concordance with their ICD-9 equivalents schizophrenia and manic-depressive psychosis, clearly shows that despite the change of diagnostic labels with time the degree of diagnostic reliability remains high. Or, the change in rates or phenomenology of the disorders cannot be simply attributed to the change in nomenclature and diagnostic criteria but is more substantial.

**Table 1:** Changes in psychopathology—key points.

Accelerated cultural transition in contemporary society, particularly in high-income countries Mass cognitive distortions Changes in normal psyche—drop of vigilance and intuitiveness, consumeristic and hedonic lifestyles, shared identities, and cognitive insecurity Reduction of mental resilience Pathomorphosis of clinical pictures, blurring of diagnostic boundaries, mimicry of psychopathology, and overdiagnosis Need for shift of mental health care focus from stressors to the host, and from protection to resilience

Some contemporary changes in psychopathology include (but are not limited to): pathomorphosis of certain clinical characteristics such as loss of the classical cyclic course of affective disorders with poor demarcation of the episodes in time and persistence of residual symptoms; rise of psychoses due to, or provoked by, substances, reciprocal to a drop of some typical schizophrenic manifestations such as the hebephrenic and catatonic forms which are increasingly rarer, especially in high-income countries; rise of personality pathology and oddities that are hard to differentiate from psychopathology; and new forms of social withdrawal such as hikikomori (16). Overdiagnosis is evident by the drop of diagnostic thresholds and expanding the diagnostic boundaries, especially of the bipolar spectrum (17), ADHD (18), and probably of the autism spectrum disorders (19). Besides its association with other cultural, research, and health care factors, the drop of diagnostic thresholds may be also connected to the drop of resilience and lowering of the threshold for experiencing distress or for dysfunction (both obligatory as diagnostic criteria for most categories in the current classifications of mental disorders), and hence—for complaining and seeking help.

## Discussion

The impact of these changes in psychopathology on mental health care is related primarily to blurring of the boundaries between norm and pathology and to gnostic relativism. The rise of narcissistic pathology and new forms of anxiety and alienation in our age correspond roughly to what the fin de siècle illness model of the classical neurosis was for the end of the nineteenth century. In the context of uncontestably positive developments such as tolerance to deviations, decreased stigma, and increased accessibility to professional help, there is nowadays a risk for neglect of severe pathology at the expense of overtreatment of sub-threshold or doubtful one. With overdiagnosis, discrimination between norm and pathology, and particularly between psychosis and non-psychosis, may become more difficult. In clinical practice, sometimes even psychotic functioning without psychosis can be observed in some alienated young persons (particularly males). And the presence of oddities without illness creates the perspective of peculiar *mimicry of psychopathology*—strange behaviors that phenomenologically imitate symptoms without being symptoms.

While it is of ultimate importance to address mental health issues in general, and in the current pandemic situation in particular (13), due attention should be given not only to agents threatening mental health from outside, but also to the host, i.e., the inner resistance strengths of the recipient. Instead of focusing primarily on protection against stressors and needs unmet by objective restrictions—thus reinforcing complaining, demoralizing, and even litigation behavioral and illness styles—it would be more constructive to invest efforts also in strengthening mental health resilience. It is wiser to increase immunity and to protect against infection than to protect only. Besides just reproducing concerns about vulnerability of mental health at the face of calamity, trying to find out why it happened to be so vulnerable and how can we improve it could prove to be more productive. Such an approach fits our professional integrity better than offering cures to healthy people.

## Author Contributions

The author confirms being the sole contributor of this work and has approved it for publication.

## Conflict of Interest

The author declares that the research was conducted in the absence of any commercial or financial relationships that could be construed as a potential conflict of interest.

## Footnotes

^1^*World Values Survey Database*. Available online at: http://www.worldvaluessurvey.org/wvs.jsp (accessed December 29, 2020).

## References

[1] Whitley R. Postmodernity and mental health. Harv Rev Psychiatry. (2008) 16:352–64. 10.1080/10673220802564186 19085389

[2] Minkov M. Cultural Differences in a Globalizing World. Bingley: Emerald (2011).

[3] Dawkins R. The Selfish Gene 30th Anniversary Edition. 3rd ed. Oxford: Oxford University Press (2006).

[4] Frankfurt H. On Bullshit. Princeton, NJ: Princeton University Press (2005).

[5] Onchev G. Culture and Psychopathology. The Anthropology of Mental Illness (Onchev G, 2017, transl. by S. Kleinsasser). Berlin, Bern, Bruxelles, New York, NY, Oxford, Warszawa, Wien: Peter Lang (2019). 10.3726/b15853

[6] Diamond J. The World Until Yesterday: What Can We Learn From Traditional Societies? New York, NY: Viking Press (2012).

[7] Young J Klosko JS Weishaar ME. Schema Therapy: A Practitioner's Guide. New York, NY: Guilford Press (2003).

[8] Blackmore S. The Mem? Machine. Oxford: Oxford University Press (1999).

[9] Abed R Brüne M. The role of the evolutionary approach in psychiatry. World Psychiatry. (2019) 18:370–1. 10.1002/wps.20688 31496100 PMC6732701

[10] World Health Organization. Preamble to the Constitution of WHO as Adopted by the International Health Conference, New York, 19 June – 22 July 1946; signed on 22 July 1946 by the Representatives of 61 States (Official Records of WHO, no. 2, p. 100) and Entered Into Force on 7 April 1948. Geneva: WHO (2014).

[11] Galderisi S Heinz A Kastrup M Beezhold J Sartorius N. Towards a new definition of mental health. World Psychiatry. (2015) 14:231–3. 10.1002/wps.20231 26043341 PMC4471980

[12] Linden D. Euthymic suffering and wisdom psychiatry. World Psychiatry. (2020) 19:55–6. 10.1002/wps.20718 31922666 PMC6953571

[13] Unützer J Kimmel RG Snowden M. Psychiatry in the age of COVID. World Psychiatry. (2020) 19:130–1. 10.1002/wps.20766 32394549 PMC7214943

[14] Paris J. Overdiagnosis in Psychiatry: How Modern Psychiatry Lost Its Way by Creating a Diagnosis for Almost All of Life's Misfortunes. London: Oxford University Press (2015). 10.1093/med/9780199350643.001.0001

[15] Jablensky A Hugler H von Cranach M Kalinov K. Kraepelin revisited: a reassessment and statistical analysis of dementia praecox and manic-depressive insanity in 1908. Psychol Med. (1993) 23:843–58. 10.1017/S0033291700026337 8134510

[16] Wu AFW Ooi J Wong PWC Catmur C Lau JYF. Evidence of pathological social withdrawal in non-Asian countries: a global health problem. Lancet Psychiatry. (2019) 6:195–6. 10.1016/S2215-0366(18)30428-0 30798886

[17] Zimmerman M Ruggero CJ Chelminski I Young D. Is bipolar disorder overdiagnosed? J Clin Psychiatry. (2008) 69:935–40. 10.4088/jcp.v69n0608 18466044

[18] Hinshaw SP Scheffler RM. The ADHD Explosion: Myths, Medication, Money, and Today's Push for Performance. New York, NY: Oxford University Press (2014).

[19] Røtgaard E-M Jensen K Vergnes J-N Soulières I Mottron L. Temporal changes in effect sizes of studies comparing individuals with and without autism: a meta-analysis. JAMA Psychiatry. (2019) 76:1124–32. 10.1001/jamapsychiatry.2019.1956 31433441 PMC6704749

